# Lea’s Series of Pocket Textbooks

**Published:** 1902-05

**Authors:** 


LEA'S SERIES OF POCKET TEXTBOOKS. Hayden on Venereal Diseases. A Pocket Text-book of Venereal Diseases. For Students and Practitioners. By JAMES R. HAYDEN, M. D., Chief of Clinic and Instructor in Venereal and Genito-Urinary Diseases in the College of Physicians and Surgeons, New York, etc. New (3d) edition, thoroughly revised. In one 12mo volume of 304 pages, with 66 engravings. Philadelphia and New York: Lea Brothers & Co. 1901. [Cloth, $1.75 net; flexible leather, $2.25 net.]

A third edition of this excellent volume should speak for itself. Still Hayden has found it necessary to make many changes from the second, which brings it practically up to date. A few points seem to have been lost sight of or not considered of sufficient importance to mention; for example, the use of leeches in acute prostatitis, resolution being brought about by these in a few hours, where days would be necessary under the advised treatment. The same may be said of silver in acute cystitis, say in 1-15,000 solutions, the patient not being confined to bed at all, while the result is about the same as in acute prostatitis.

In the treatment of chronic gonorrhea the author still clings to the use of the catheter for introducing the solution. It is the reviewer's opinion that this is entirely unnecessary, only producing irritation, while the solution does not distend the canal the same as though introduced from the meatus with the syringe as the author advises. Hayden must be disagreed with in the opinion that filling the urethra from the meatus does not distend the entire anterior canal, and if used carefully no traumatism of any account will result. Furthermore, it is the reviewer's findings that even eight ounces of urine will not thoroughly cleanse the canal of all adherent pus and débris, which if allowed to remain is a most serious obstacle in that it is loaded with pyogenic bacteria that cannot but be forced into the many sinuses of the prostate, thus tending to increase the trouble. If preinstrumental flushing was practised in every case there would be less follicular prostatitis, epididymitis and the like. On the whole, however, no more valuable work on the subject with which it deals has been published in recent years. J. H. D.

